# Association of serum sclerostin levels with marrow adiposity in postmenopausal women with glucocorticoid-induced osteoporosis

**DOI:** 10.1186/s12902-024-01591-8

**Published:** 2024-04-28

**Authors:** Wei Li, Wei Wang, Minlan Zhang, Qi Chen, Fengyi Li, Shaojun Li

**Affiliations:** 1 Department of Radiology, Pudong New Area, Shanghai University of Medicine & Health Sciences Affiliated Zhoupu Hospital, No. 1500 Zhouyuan Road, Shanghai, 201318 China; 2 Department of Laboratory Medicine, Pudong New Area, Shanghai University of Medicine & Health Sciences Affiliated Zhoupu Hospital, Shanghai, 201318 China

**Keywords:** Glucocorticoid-induced osteoporosis, Sclerostin, Marrow adiposity, Magnetic resonance spectroscopy

## Abstract

**Background:**

Glucocorticoids and sclerostin act as inhibitors of the Wnt signaling pathway, thereby hindering bone formation. Given the pathway's intricate association with mesenchymal stem cells, the hypothesis suggests that heightened sclerostin levels may be intricately linked to an augmentation in marrow adiposity induced by glucocorticoids. This study endeavored to delve into the nuanced relationship between circulating sclerostin and bone marrow adipose tissue in postmenopausal women grappling with glucocorticoid-induced osteoporosis (GIO).

**Methods:**

In this cross-sectional study, 103 patients with autoimmune-associated diseases underwent glucocorticoid treatment, boasting an average age of 61.3 years (standard deviation 7.1 years). The investigation encompassed a thorough assessment, incorporating medical history, anthropometric data, biochemical analysis, and dual-energy X-ray absorptiometry measurements of lumbar and femoral bone mineral density (BMD). Osteoporosis criteria were established at a T-score of -2.5 or lower. Additionally, MR spectroscopy quantified the vertebral marrow fat fraction.

**Results:**

BMD at the femoral neck, total hip, and lumbar spine showcased an inverse correlation with marrow fat fraction (*r* = –0.511 to – 0.647, *P* < 0.001). Serum sclerostin levels exhibited a positive correlation with BMD at various skeletal sites (*r* = 0.476 to 0.589, *P* < 0.001). A noteworthy correlation emerged between circulating sclerostin and marrow fat fraction at the lumbar spine (*r* = –0.731, 95% CI, –0.810 to –0.627, *P* < 0.001). Multivariate analysis brought to light that vertebral marrow fat fraction significantly contributed to sclerostin serum concentrations (standardized regression coefficient ß = 0.462, *P* < 0.001). Even after adjusting for age, body mass index, physical activity, renal function, BMD, and the duration and doses of glucocorticoid treatment, serum sclerostin levels maintained a significant correlation with marrow fat fraction.

**Conclusions:**

Circulating sclerostin levels exhibited a noteworthy association with marrow adiposity in postmenopausal women grappling with GIO.

## Introduction

Sclerostin, derived from osteocytes, navigates through canalicular networks to reach bone surfaces. Upon reaching these surfaces, sclerostin impedes the canonical Wnt-beta-catenin pathway, thereby reducing osteoblastogenesis and accelerating osteoclastogenesis [1–3]. In 2019, the European Medicines Agency approved romosozumab, an anti-sclerostin antibody, for osteoporosis treatment [4].

The regulatory influence of the Wnt pathway intricately shapes the differentiation process of bone marrow mesenchymal stem cells (BMSCs) [5]. Glucocorticoids promote the differentiation of BMSCs towards adipocytes rather than osteoblasts, and both endogenous and exogenous glucocorticoids may induce the accumulation of bone marrow adipose tissue (BMAT) [6, 7]. Central to the pathogenesis of glucocorticoid-induced osteoporosis (GIO) is the suppression of bone formation. One of the key mechanisms by which glucocorticoids diminish the production of osteoblasts is through the antagonism of the Wnt/β-catenin signaling pathway [8]. Additionally, sclerostin acts as a Wnt signaling inhibitor, obstructing osteogenic differentiation and impeding the acquisition of bone mass [3, 8, 9]. Fascinatingly, treatments with sclerostin-neutralizing antibodies demonstrated the ability to reduce overall marrow adiposity in both a time-dependent and dose-dependent fashion [5]. Furthermore, a discernible negative correlation was observed between the areas of bone and BMAT [10]. This suggests that elevated sclerostin levels may be associated with increased bone marrow adiposity induced by glucocorticoids.

Currently, our comprehension of the intricate relationships involving skeletal integrity, marrow fat, body compositions, and the impact of circulating sclerostin on these dynamics is notably limited, further complicated by conflicting findings reported in the existing literature [1, 5, 11–13]. Sclerostin levels rise in the serum of mice subjected to both synthetic glucocorticoid treatment and exposure to endogenous glucocorticoids. However, the regulation of its expression in bone tissue or murine BMSCs treated with glucocorticoids remains ambiguous. In contrast, sclerostin levels decrease in human BMSCs when stimulated with glucocorticoids and in patients with rheumatoid arthritis and polymyalgia rheumatica who necessitate glucocorticoid therapy [14]. Interestingly, other reports have demonstrated elevated serum levels of sclerostin in patients undergoing glucocorticoid therapy [15]. Furthermore, no association was found between circulating sclerostin levels and bone marrow adiposity in postmenopausal women with osteoarthritis [1]. However, comprehensive data on circulating sclerostin levels, particularly its correlation with marrow adiposity in postmenopausal women with GIO, is limited.

The primary aim of this study was to explore the potential association between circulating sclerostin levels and BMAT assessed by MR spectroscopy in postmenopausal women with GIO.

## Methods

### Ethics approval and informed consent

The research protocol underwent a comprehensive review and obtained approval from the Institutional Review Board at Zhoupu Hospital, affiliated with Shanghai University of Medicine & Health Sciences. Before participating in the study, all participants provided written informed consent.

### Study population

We undertook a cross-sectional investigation that involved consecutive postmenopausal females undergoing chronic glucocorticoid treatment, defined as a daily dose of ≥ 5 mg of prednisone or its equivalent for a duration exceeding 3 months [16]. Patients enrolled in this study were referred to our unit between January 2018 and December 2023, with a primary emphasis on assessing bone metabolism. At the study's onset, each participant underwent a comprehensive clinical visit and a thorough laboratory evaluation. Major exclusion criteria included: (1) the use of anti-osteoporotic drugs at any point, including bisphosphonates, hormone replacement therapy, parathyroid hormone, selective estrogen receptor modulators, etc.; (2) a diagnosis of metabolic bone disease unrelated to GIO; (3) chronic kidney disease with calculated creatinine clearance < 30 mL/min; (4) contraindication for magnetic resonance imaging. Additionally, other conditions impeding study participation, such as mental illness, were considered as exclusion criteria. A total of 103 postmenopausal women who met the inclusion criteria were considered for the final analysis.

In the process of gathering our data, we systematically compiled a comprehensive array of clinical information. This encompassed a spectrum of demographic details, including age, gender, menopausal status, and individual health status. Our focus extended to a meticulous examination of medication history, with a particular emphasis on factors contributing to osteoporosis risk, such as glucocorticoid doses—including the daily current dose, duration, and cumulative dose in prednisone equivalent. We also delved into the use of additional immunosuppressant agents and explored diverse lifestyle factors. This encompassed intricate aspects like dietary habits, including dairy consumption, the usage of vitamins or calcium supplements, as well as behaviors like cigarette smoking and alcohol intake. Furthermore, anthropometric data, comprising height, weight, and body mass index (BMI) measured in kilograms per square meter, were meticulously recorded. Physical activity was precisely defined as active participation in exercise sessions occurring at least three times a week, each lasting a minimum of 30 min.

### Laboratory analyses

Blood samples were meticulously collected between 8 and 9 a.m. following an overnight fast, a precautionary measure aimed at minimizing potential confounding factors and ensuring the precise assessment of bone health. Utilizing enzymatic methods (ADVIA Chemistry XPT; SIEMENS, German), we conducted a thorough analysis, encompassing the measurement of serum uric acid, creatinine, phosphorus, calcium, and the serum lipid profile, which included total cholesterol, high-density lipoprotein cholesterol, low-density lipoprotein cholesterol, and triglyceride levels, as well as fasting blood glucose. The estimated glomerular filtration rate (eGFR) was calculated using the CKD-EPI formula (mL/min). For the evaluation of markers of bone turnover, serum was separated by centrifugation and kept at − 20 °C for the determination of parathyroid hormone, c-terminal telopeptide, total osteocalcin, intact N-terminal propeptide of type I collagen, 25-hydroxyvitamin D, sclerostin and osteocalcin using electrochemiluminescence assays (Cobas e601; Roche Diagnostics, Basel, Switzerland). Additionally, plasma glycated hemoglobin levels were determined with high-performance liquid chromatography (MQ-6000; Shanghai Medconn Biotechnology Co., Ltd., China).

### Dual-energy X-ray absorptiometry

The assessment of areal bone mineral density (BMD, g/cm^2^) at the lumbar spine and proximal femur (femoral neck and total hip) is a pivotal marker for gauging bone health, a task commonly executed through dual-energy X-ray absorptiometry (DXA, GE Healthcare, WI, USA). Renowned for its precision and steadfastness in BMD measurements, this machine enjoys widespread adoption in the field. In order to safeguard the credibility of our DXA scans, our center strictly adheres to rigorous quality control protocols. A crucial determinant of reliability is the coefficient of variation. In our study, the coefficients of variation for total femur and lumbar spine BMD measurements was 1.40% and 1.18%, respectively, underscoring the robustness and precision of our assessments. Densitometric osteoporosis was defined according to the WHO criteria with T–score values ≤ –2.5 (in subjects > 50 years) or Z–score values < –2 (in subjects < 50 years) [16].

### MR spectroscopy acquisition and quantification

We employed MR spectroscopy to precisely assess the fat content within the vertebral marrow. The MRI procedures were executed on a 3-T clinical system (MAGNETOM Skyra; Siemens Medical Systems, Erlangen, Germany). A surface coil was strategically placed as the radiofrequency receiver beneath the lumbar spine region, while the body volume coil served as the radiofrequency transmitter. Participants assumed a supine position within the scanner to ensure the accuracy of imaging. In order to cater to individual clinical needs, a standard clinical MRI of the lumbar spine was conducted. This thorough examination encompassed sagittal turbo-spin echo T1- and T2-weighted imaging, along with a transaxial T2-weighted fast spin echo sequence. Sagittal images of the lumbar spine were acquired to facilitate the precise positioning of the L3 vertebral body. Following this, a stimulated echo acquisition method (STEAM) sequence was implemented for MR spectroscopy to ascertain the percentage of bone marrow fat.

In the realm of lumbar MR spectroscopy, a precisely defined voxel measuring 1.5 cm × 1.5 cm × 1.5 cm was strategically positioned within the intricate confines of the L3 vertebral body. The volume of interest, meticulously centered in the middle of the vertebral body, maintained unwavering dimensions throughout the entirety of the study. A region-specific STEAM (stimulated echo acquisition mode) sequence was employed for MR spectroscopy. Water suppression was not implemented during the acquisition process. The sequence parameters were meticulously configured as follows: repetition time = 3000 ms, echo times) = 12, 24, 36, 48, and 72 ms, data points = 1028, and receiver bandwidth = 2000 Hz. Automated procedures were adeptly employed for gradient shimming and the fine-tuning of transmit and receive gain. At each echo time, the signal intensities of the distinct fat and water peaks were precisely measured through integration of their corresponding spectral regions. Following a meticulous consideration of T2 decay, accomplished by fitting an exponential function to the signal evolution across echo time, the precise determination of fat percentage unfolded with exactitude. This intricate calculation relied on the scrupulous measurement of signal intensities linked to both fat and water [17, 18], as impeccably illustrated in Supplementary Material Fig. 1.

**Fig. 1:**
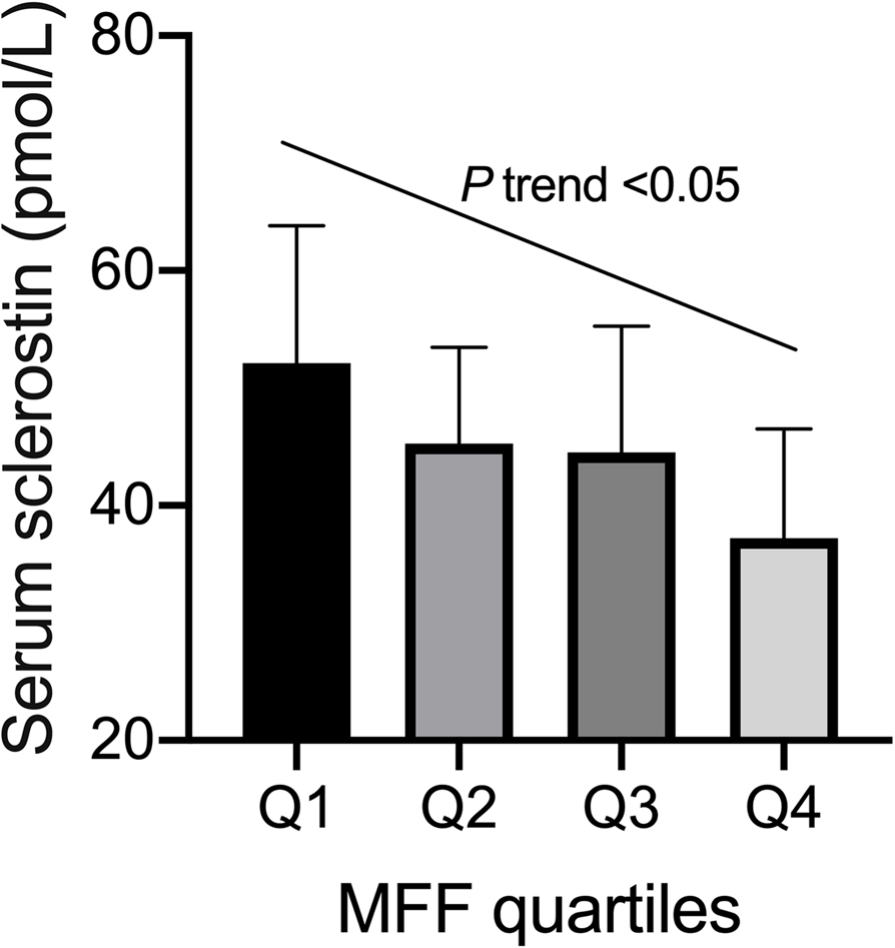
Serum sclerostin levels according to degree of marrow fat fraction (MFF) given in quartiles. Trend analysis was performed for serum sclerostin levels to describe a linear component of the trend from quartile 1 (Q1) to quartile 4 (Q4) of the vertebral MFF adjusted for age, body mass index, physical activity, renal function, bone mineral density, and the duration and doses of glucocorticoids treatment. MFF quartiles: Q1 < 56.0% (*n* = 25); 56.0% ≤ Q2 < 60.5% (*n* = 26); 60.5% ≤ Q3 < 69.0% (*n* = 26); and 69.0% ≤ Q4 (*n* = 26). Error bars represent standard deviation values of the mean

### Statistical analysis

The descriptive statistics included in the analysis were the mean ± standard deviation (SD), median values with interquartile ranges (IQ1-IQ3), counts, and frequencies. The Shapiro-Wilks test was performed to estimate the normal distribution of all outcomes. The association between variables was assessed using Pearson’s or Spearman’s rank test, as appropriate. Serum sclerostin levels were subjected to trend analysis to reveal the linear progression across quartile groups (Q1 to Q4), determined by the distribution of marrow fat fraction in quartiles. The impact of independent variables on serum sclerostin levels was evaluated through a multiple regression analysis using the ordinal least squares method. Models were adjusted for age, BMI, physical activity, renal function, BMD, and the duration and doses of glucocorticoids treatment as previous studies have reported associations with sclerostin levels [11, 19–21]. A *p*-value of < 0.05 was considered statistically significant. The statistical analysis was performed with SPSS version 25.0 (IBM Corp., SPSS Statistics, Armonk, NY, USA).

## Results

### Baseline characteristics

A total of 103 postmenopausal women met the inclusion criteria, with a mean age of 61.3 years (SD 7.1 years) spanning from 50 to 75 years, were undergoing chronic treatment with glucocorticoids. The median duration and doses of glucocorticoids treatment were calculated at 14.5 months (IQR: 8.3, 27.8 months) and 8 mg/day (IQR: 6, 10 mg/day), respectively. The primary autoimmune-associated diseases prevalent among the participants including rheumatoid arthritis (*n* = 70), polymyalgia rheumatica (*n* = 19), vasculitis (*n* = 4), systemic lupus erythematosus (*n* = 5), inflammatory myopathies (*n* = 3), and collagenosis (*n* = 2). For a comprehensive overview of the clinical characteristics of the patients, refer to Table 1.

**Table 1:** Summary of demographic and clinical characteristics included in the study

Parameters	Cases (*n* = 103)
Body mass index (kg/m^2^)	24.1 ± 3.2
Current smoking, *n* (%)	10 (9.7%)
Alcohol drinkers, *n* (%)	12 (11.7%)
Physical activity, *n* (%)	38 (36.9%)
Fasting blood glucose (mmol/L)	4.8 ± 1.2
HbA1C (%)	5.0 ± 0.7
Total cholesterol(mmol/L)	4.83 ± 0.97
Triglycerides (mmol/L)	0.62 ± 0.25
HDL-C(mmol/L)	1.60 ± 0.43
LDL-C (mmol/L)	2.47 ± 0.59
25-hydroxyvitamin D (ng/ml)	20.1 ± 4.6
PINP (ng/ml)	33.5 ± 5.7
Osteocalcin (ng/ml)	14.3 ± 3.8
CTX (ng/ml)	1.55 ± 0.59
Sclerostin (pmol/L)	44.7 ± 7.5
Lumbar spine BMD (g/cm^2^)	0.830 ± 0.101
Total hip BMD (g/cm^2^)	0.738 ± 0.091
Femoral neck BMD (g/cm^2^)	0.714 ± 0.085
Marrow fat fraction (%)	62.3 ± 9.5

Data are presented as mean ± standard deviation or *n* (%)

*BMD* bone mineral density, *CTX* c-terminal telopeptide, *HbA1c* hemoglobin A1c, *HDL-C* high-density lipoprotein cholesterol, *LDL-C* low-density lipoprotein cholesterol, *PINP* intact N-terminal propeptide of type I collagen

### Associations between serum sclerostin and parameters of interest

Table 2 delineates the correlations between serum sclerostin and key parameters. Positive correlations emerged between circulating sclerostin and BMD at the femoral neck, total hip, and lumbar spine in postmenopausal women with GIO. Additionally, BMD at the femoral neck, total hip, and lumbar spine exhibited an inverse correlation with marrow fat fraction. Noteworthy is the significant negative correlation identified between sclerostin serum concentrations and eGFR, as well as between marrow fat fraction and eGFR, suggesting higher serum sclerostin levels or elevated marrow adiposity in individuals with lower eGFR. No significant correlations were observed between age, markers of bone turnover, and circulating sclerostin or marrow fat fraction.

**Table 2:** Correlations between circulating sclerostin levels and the parameters of interest

Variables	Sclerostin (pmol/L)		Marrow fat fraction (%)	
	*r*	*p*-values	*r*	*p*-values
Lumbar spine BMD (g/cm^2^)	0.589	< 0.001	– 0.647	< 0.001
Femoral neck BMD (g/cm^2^)	0.476	< 0.001	– 0.511	< 0.001
Total hip BMD (g/cm^2^)	0.503	< 0.001	– 0.544	< 0.001
eGFR (mL/min)	– 0.284	0.015	– 0.305	0.009
Age (years)	0.097	0.400	0.109	0.313
PINP (ng/ml)	0.024	0.602	– 0.072	0.574
Osteocalcin (ng/ml)	0.081	0.417	0.035	0.832
CTX (ng/ml)	– 0.109	0.703	0.046	0.740

*BMD* bone mineral density, *CTX* c-terminal telopeptide,* eGFR* estimated glomerular filtration rate, *PINP* intact N-terminal propeptide of type I collagen

### Associations between circulating sclerostin and marrow fat fraction

**Fig. 2:**
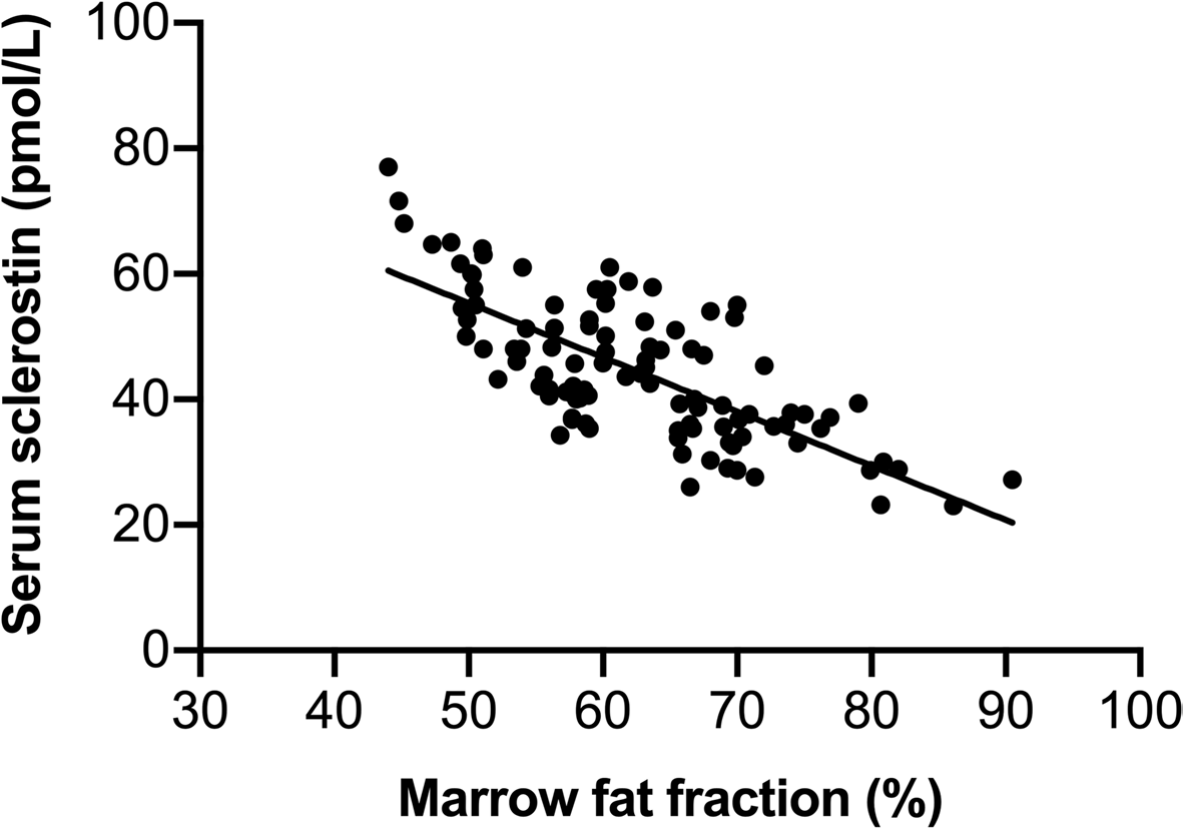
Relationship between serum sclerostin levels and marrow fat fration (*r* = –0.731, 95% CI, –0.810 to –0.627, *P* < 0.001)

As depicted in Fig. 1, serum sclerostin levels exhibited a noteworthy decrease in higher vertebral PDFF quartiles compared to lower quartiles (*P* for trend < 0.05), with adjustments made for age, BMI, physical activity, renal function, BMD, and the duration and doses of glucocorticoids treatment. A significant correlation was identified between circulating sclerostin and marrow fat fraction at the lumbar spine (*r* = –0.731, 95% CI, –0.810 to –0.627, *P* < 0.001) (Fig. 2). Moreover, in the multiple regression analysis, vertebral marrow fat fraction significantly contributed to sclerostin serum concentrations (standardized regression coefficient ß = 0.462, *P* < 0.001), even after accounting for age, BMI, physical activity, renal function, BMD, and the duration and doses of glucocorticoids treatment.

## Discussion

Sclerostin, a glycoprotein, plays a pivotal role in impeding the processes associated with bone formation and stands as a key factor in diminishing osteoblast function. Additionally, sclerostin upregulates the expression of proteolytic enzymes in osteoclasts [22]. Several studies have demonstrated a direct association between BMD and sclerostin concentration in both healthy humans and individuals with spinal cord injuries [23–25]. Consistent with the findings in patients with a fresh hip fracture [26] or hematological disorders undergoing 1-year glucocorticoid therapy (> 7.5 mg/day) [15], as well as in mice exposed to endogenous and exogenous glucocorticoids [14], we identified significant positive correlations between circulating sclerostin levels and BMD measurements in postmenopausal females with GIO. These studies suggest that sclerostin concentration might serve as a complementary or potentially alternative indicator to densitometric testing [2, 23, 24]. One suggested rationale for this positive association between sclerostin and BMD posits that circulating sclerostin levels are contingent upon the number and activity of osteocytes, theoretically correlating with the overall bone mass [26]. Elevated BMD might lead to an increased number of osteocytes, consequently resulting in higher levels of circulating sclerostin.

Nevertheless, Patalong-Wojcik and collaborators found no discernible disparity in sclerostin levels between groups with normal and diminished BMD. Moreover, there was an absence of correlation between sclerostin concentration and lumbar vertebrae BMD in young adult women [2]. The observed disparity could potentially be attributed to variations in population characteristics, including factors such as gender, menopausal status, and notably, the age range of the study cohort.

Descriptive studies involving both humans and animals have been conducted on serum sclerostin, with a primary focus on assessing skeletal integrity and bone marrow adipose tissue [1, 2, 5, 11, 13–15, 23–33]. The results are presented in Table 3. A few studies suggested that sclerostin may be associated with marrow adiposity [5, 11]. Ma et al. reported that vertebral marrow adiposity was greater in elderly men with higher serum sclerostin levels in models adjusted for age, BMI and diabetes, but not in women [11]. There were a few limitations to that study, such as the age of the population, which included only older white adults (mean age 79 years). Additionally, the factors used for adjustment were different from those used in our study and did not include eGFR, BMD, and the duration and doses of glucocorticoids treatment, which may have influenced the results. However, the complex interplay between the marrow adipose depot and osteocytes, along with osteocyte-derived molecules like sclerostin, remains largely unexplored. Fairfield and colleagues shed light on this relationship by demonstrating that sclerostin not only promotes adipocyte differentiation in pre-adipocyte cell lines but also in primary MSCs obtained from both mouse and human, exerting its effects at the expense of osteoblastogenesis. Reinforcing their findings with two in vivo models designed to inhibit sclerostin, the research unequivocally affirms that the induction of BMAT is orchestrated by sclerostin and is diminished in the absence of sclerostin, achieved through either knockout or neutralization using a specific antibody [5, 10].

However, others reported that no substantial correlations were found between circulating sclerostin and marrow proton density fat fraction in the lumbar spine, femoral neck, and femoral diaphysis in both postmenopausal women with fragility fractures and controls (postmenopausal women with osteoarthritis). This lack of correlation persisted both before and after accounting for factors such as age, eGFR, and BMD [1].

The correlation between vertebral marrow fat fraction in our study cohort and BMD values demonstrated an inverse relationship, consistent with previously published data highlighting the negative association between bone marrow adipocytes and BMD in GIO [6]. While numerous studies, particularly those conducted clinically and in vivo with rodents, have highlighted an augmentation in bone marrow adipose tissue in response to glucocorticoids [6, 34–36], it's crucial to note that this phenomenon is not universally observed. For instance, a study on outbred Swiss mice, exposed to short-term glucocorticoid excess at two different doses (2.8 and 5.4 mg/kg/d), revealed no significant impact on adipocyte volume density. This density was estimated by dividing the total number of points hitting adipocytes within the bone marrow by the total number of points hitting the bone marrow. Surprisingly, only the higher dose of glucocorticoids demonstrated a substantial increase (+ 48%) in adipocyte cross-sectional area compared to the placebo pellet [37]. The variations observed in these research findings could be attributed to a spectrum of factors, encompassing fluctuating glucocorticoid dosages, durations of drug intervention, the inclusion of diverse rodent species, the methodologies applied in measuring adipose tissue, and discrepancies in skeletal locations.

The primary discovery from our study reveals a negative association between sclerostin serum concentrations and marrow adiposity in postmenopausal females with GIO. In the literature, conflicting findings arise when investigating the regulation of sclerostin by glucocorticoids. Several previous studies have indicated that both the administration of synthetic glucocorticoids and exposure to endogenous glucocorticoids led to elevated systemic sclerostin levels in mice [14, 38]. Intriguingly, in animal models, skeletal sclerostin expression either witnessed an increase [39] or displayed no discernible difference [14, 40] following glucocorticoid treatment.

Differing from observations in animal studies, the regulation of sclerostin in the human system demonstrated increased consistency, marked by a reduction observed after glucocorticoid treatment in human bone marrow stromal cells. Additionally, patients undergoing glucocorticoid therapy for conditions such as rheumatoid arthritis, polymyalgia rheumatica and chronic inflammatory diseases exhibited a noteworthy decrease in sclerostin levels [14, 28, 29]. The regulation of sclerostin shows variations in individuals with hypercortisolism. One study documented reduced sclerostin levels in those with endogenous hypercortisolism compared to healthy controls [30], whereas another reported increased sclerostin levels [31, 41]. The contrasting results could be linked to differing methodologies, including variations in the timing of blood sample collection, the differentiation between non-fasting and fasting blood samples, discrepancies in study populations, or differences in the methods used to assess blood sclerostin levels.

Despite its strengths, our study has limitations, primarily associated with its cross-sectional nature and the absence of a designated control group. Examining the temporal correlation between circulating sclerostin and BMAT proved impractical. Additionally, our study focused solely on postmenopausal women aged 50 years and older, potentially constraining the applicability of our findings to younger women or men. Notable strengths, however, include the comprehensive assessment of marrow fat across all subjects in the study and the homogeneity of the patient group, as all individuals were undergoing chronic glucocorticoid treatment with a daily dose of ≥ 5 mg. These factors contribute to the robustness of our study.

In conclusion, we have established a correlation between circulating sclerostin levels and marrow adiposity in postmenopausal females with GIO. These findings imply that sclerostin may play significant roles in the pathogenesis of GIO.

## Data Availability

The datasets generated during and/or analysed during the current study are not publicly available but are available from the corresponding author on reasonable request.

## Abbreviations

BMAT: Bone marrow adipose tissue
BMD: Bone mineral density
BMI: Body mass index
BMSCs: Bone marrow mesenchymal stem cells
eGFR: Stimated glomerular filtration rate
GIO: Glucocorticoid-induced osteoporosis
